# Validating genetic variants in innate immunity linked to infectious events in acute myeloid leukemia post-induction chemotherapy

**DOI:** 10.1038/s41435-024-00285-4

**Published:** 2024-07-09

**Authors:** Ulf Schnetzke, Mike Fischer, Christoph Röllig, André Scherag, Heidi Altmann, Friedrich Stölzel, Nael Alakel, Martin Bornhäuser, Andreas Hochhaus, Sebastian Scholl

**Affiliations:** 1 Klinik für Innere Medizin II, Abteilung für Hämatologie und Internistische Onkologie, Comprehensive Cancer Center Central Germany – Campus Jena, Universitätsklinikum Jena, Jena, Germany; 2 Institut für Humangenetik, Universitätsklinikum Jena, Jena, Germany; 3 Medizinische Klinik I, Universitätsklinikum der Technischen Universität Dresden, Dresden, Germany; 4 Institut für Medizinische Statistik, Informatik und Datenwissenschaften, Universitätsklinikum Jena, Jena, Germany; 5 Sektion für Stammzelltransplantation und zelluläre Immuntherapie, Klinik für Innere Medizin II, Abteilung für Hämatologie und Onkologie, Universitätsklinikum Schleswig-Holstein, Campus Kiel, Christian-Albrechts-Universität Kiel, Kiel, Germany

**Keywords:** Infection, Pattern recognition receptors

## Abstract

Infectious events, such as sepsis and invasive fungal disease (IFD), pose significant risks in patients with acute myeloid leukemia (AML). Previous studies, including our own, have suggested a potential role of single nucleotide polymorphisms (SNPs) within the innate immune system in influencing individual infection susceptibility. However, many of these associations lack validation in independent cohorts. This study sought to validate the impact of 11 candidate SNPs across 6 genes (*TLR2, TLR4, Dectin-1, DC-SIGN, PTX3, L-Ficolin*) in an independent cohort of patients. Two cohorts with newly diagnosed AML patients receiving intensive induction chemotherapy were analyzed: a stratification cohort comprising 186 patients and a validation cohort consisting of 138 patients. Multiple SNPs in each cohort were found to be associated to infectious complications, notably the *DC-SIGN* SNP rs4804800 demonstrated a significant association with sepsis in both cohorts. SNPs within the *PTX3* and Dectin-1 genes were linked to IFD development in one cohort each. This study represents the first validation study of candidate genes associated with infectious events in AML patients after intensive induction chemotherapy. Identifying genetic predispositions to infections could significantly impact the management of antimicrobial prophylaxis and treatment in AML patients.

## Introduction

Infectious complications remain a significant cause of morbidity and mortality in patients undergoing curative-intent induction chemotherapy for acute myeloid leukemia (AML). During recent years remarkable advances have been made in supportive therapy especially in infectious prophylaxis. Still, a relevant proportion of patients experience septic complications most often due to pneumonia or blood stream infections [1–3]. Patient specific individual genetic alterations affecting the immune response are identified to contribute to susceptibility to infectious complications [4, 5]. Induction chemotherapy in patients with newly diagnosed AML resulting in a long lasting neutropenic period might act as a potent model for susceptibility of the innate immune system [6]. We and others reported different single nucleotide polymorphisms (SNPs) and their association with the occurrence of infectious diseases in AML patients. In summary, within a cohort of AML patients following induction chemotherapy, it was observed that polymorphisms of *TLR2* and *TLR4* influence the risk of developing sepsis and pneumonia [7]. Additionally, an association between polymorphisms of *Dectin-1* and *TLR2* and the occurrence of invasive fungal disease (IFD) was identified [8]. The identified genes and genetic variations were found to contribute to general immune reactivity and *Aspergillus* specific responses for IFD. Mostly, those analyses were performed within the setting of allogeneic stem-cell transplantation (alloHSCT). Furthermore, only a minority of these studies were validated independently potentially explaining discrepancies between different studies. Two studies identified and validated SNPs in *Pentraxin 3* (*PTX3*) (rs2305619/rs1840690, rs3816527) and *Dectin-1* (Y238X, rs16910526) genes that were associated with development of IFD in alloHSCT recipients [9, 10]. An impaired anti-fungal immune response has been described for both genetic polymorphisms [10–13]. The C-type lectin receptor Dectin-1 plays a pivotal role in antifungal immunity*. Dectin-1* Y238X represents a polymorphism resulting in an early stop codon leading to diminished Dectin-1 receptor activity [12]. As a consequence, a reduced ß-glucan recognition leads to a defective cytokine production and antifungal immunity on receptor engagement [11, 14, 15]. Inconclusive data about the impact of the *PTX3* SNPs rs2305619/rs1840690 and rs3816527 on the occurrence of infectious diseases exist. Cunha et al. reported that the GG phenotype of the *PTX3* SNPs rs2305619/rs1840690 and AA genotype of the of the *PTX3* SNP rs3816527, respectively, were associated with an increased risk of IFD following alloHSCT [10]. Another group showed that patients with the AA genotype of SNP rs2305619/rs1840690 have a higher susceptibility for pulmonary aspergillosis [16]. Since the alloHSCT setting cannot be translated into the chemotherapy-associated neutropenic period following induction chemotherapy clinical data on the impact of such polymorphisms and the occurrence of infectious diseases in a non-transplant situation are warranted. Following induction chemotherapy, a longstanding severe neutropenia of three to four weeks predisposes for infectious complications. In the vast majority of patients, at least one episode of febrile neutropenia is observed [17]. By including all-category IFDs the incidence is reported up to 30% following induction chemotherapy [18–20]. Even the occurrence of only probable/proven IFD has been documented in 17% of patients during first induction chemotherapy [21]. Other common causes are blood stream infections or catheter related infections [22]. Several studies link genetic variations in *DC-SIGN* (dendritic cell – specific intercellular adhesion molecule 3 – grabbing nonintegrin) to various infectious diseases [23–25]. *DC-SIGN* is a C-type lectin receptor encoded by the CD209 gene and found on the surface of both macrophages and dendritic cells [26, 27]. As a pathogen-recognition receptor, *DC-SIGN* recognizes a wide range of microorganisms such as human immunodeficiency virus 1 (HIV-1), Hepatitis C virus (HCV), SARS coronavirus or bacteria such as Mycobacterium tuberculosis [28–31].

Here, we addressed a key limitation of prior studies by independently validating SNPs that were associated with infectious events in a second cohort of patients also undergoing induction chemotherapy for AML. This study provides the initial validation of SNPs previously reported and their association with infectious complications [7, 8]. Moreover, additional SNPs were examined regarding their influence on the occurrence of infections in AML patients after induction chemotherapy.

## Materials and methods

### Study cohorts

Two independent patient cohorts of adult AML patients have been investigated. The first cohort (stratification cohort) consists of 186 Caucasian patients (83 male, 103 female; median age 58 years, range 19-78 years) with newly diagnosed AML (excluding acute promyelocytic leukemia). All patients were diagnosed at the Jena University Hospital, Germany and treated using single induction chemotherapy according to the *East German Study Group Hematology and Oncology* (OSHO) protocol consisting of intermediate dose cytarabine and mitoxantrone between the years 2003 and 2017. The second cohort (validation cohort) consists of 138 adult patients (81 male, 57 female; median age 56.5 years, range 18–76 years) with newly diagnosed AML who underwent “7 plus 3” induction chemotherapy with cytarabine and daunorubicin between the years 2012 and 2019 at the Dresden University Hospital (*German Study Alliance Leukemia*, SAL). All patients received either trimethoprim-sulfamethoxazole or ciprofloxacin and fluconazole or posaconazole for antibiotic and antifungal prophylaxis, respectively. In detail, 51% of patients received fluconazole and 49% posaconazole in the stratification cohort, whereas all patients in the validation cohort received posaconazole.

Clinical data of both cohorts are presented in Table 1. The observational period was defined from start of induction chemotherapy until discharge from hospital after hematologic reconstitution.

**Table 1:** Patients and clinical characteristics.

	Stratification cohort (*n* = 186)	validation cohort (*n* = 138)	*p* value
Median age (years, range)	57.5 (19–78)	56.5 (18–76)	n.s.
Male sex, *n* (%)	83 (45)	81 (58.7)	n.s.
Type of acute myeloid leukemia			
De novo*, n* (%)	118 (63.4)	106 (76.8)	n.s.
Secondary or therapy related, *n* (%)	68 (36.6)	32 (23.2)	n.s.
Cytogenetic risk group			
Low, *n* (%)	20 (10.8)	12 (8.7)	n.s.
Intermediate, *n* (%)	106 (57)	99 (71.7)	n.s.
Poor, *n* (%)	58 (31.2)	27 (19.6)	n.s.
WBC at diagnosis (/nl), median (range)	14.2 (0.3–330)	11.4 (0.3–273)	n.s.

*WBC* white blood count, *n.s.* not statistically significant.

Informed written consent for the project – including genetic analyses – was obtained from all patients in accordance with the current version of the Declaration of Helsinki.

### Diagnostic criteria of infectious events and pneumonia

Clinical events such as sepsis, systemic inflammatory response syndrome (SIRS) and fever of unknown origin (FUO) were defined according to the consensus definitions for sepsis [32]. The definition of sepsis also included neutropenic fever with pneumogenic focus.

Pneumonia was defined as a new infiltrate on chest X-ray or computed tomography in combination with at least two of the following criteria: cough, sputum production, temperature >38 °C or <35 °C, hemoptysis, thoracic pain or auscultatory findings consistent with pneumonia. IFD was diagnosed based upon the criteria reported by the European Organization for Research and Treatment of Cancer/Invasive Fungal Infections Cooperative Group and the National Institute of Allergy and Infectious Diseases Mycoses Study Group (EORTC/MSG) in 2008 [33].

### Analysis of polymorphisms

Samples analyzed were obtained from the biobank of the University Hospital Jena and the AML-biobank of the Study Alliance Leukemia (EK 98032010). All patients consented to biobanking and sample use for research projects. The samples both of the stratification and validation cohort were taken from patients with AML at initial diagnosis, isolated from bone marrow aspirates using a Ficoll gradient (GE Healthcare) and cryopreserved.

The detection of SNP alleles was examined using commercially available TaqMan™ SNP Genotyping Assays (Thermo Fisher Scientific, Waltham, Massachusetts, USA) on the PCR Systems 9700HT and QuantStudio 5 (Thermo Fisher Scientific), following the manufacturer’s instructions. Genotyping analysis was conducted using the cloud-based Applied Biosystems™ Analysis Software, specifically the Genotyping Analysis Module (Thermo Fisher Scientific). Additionally, the genotyping results for the *PTX3* SNPs rs3816527 and rs2305619 were verified by Sanger sequencing. Furthermore, 3 SNPs were determined using alternative genotyping methods: For the samples of the first cohort from Jena, genotyping of *TLR2* Arg753Gln (R753Q, rs5743708) and *TLR4* Thr399Ile (T399I, rs4986791) was performed by pyrosequencing as described elsewhere [7]. Genotyping of *Dectin-1* Y238X (rs16910526) was carried out using a Bi-PASA PCR method [4].

### Statistics

Standard descriptive statistics were used to summarize the data (continuous variables: quartiles or mean ± SD)/count data: absolute and relative frequencies). For the association of SNP alleles with the three outcomes (occurrence of sepsis, pneumonia or *possible* IFD) univariate and multiple logistic regression analyses were applied. For each candidate SNP locus, allelic and genotypic (additive, recessive and dominant) associations were assessed. Adjustment in multiple logistic regression was done including sex, age (linear), subtype of AML (de novo and secondary, respectively), cytogenetic risk group according to ELN 2017 criteria (3 groups) and leukocyte count (linear). Results of multiple logistic regression analyses are presented as odds ratio (OR) with 95% confidence interval (95%-CI) and corresponding two-sided, unadjusted *p* values. The level of significance was set at 5%. For statistical calculations the SPSS software package, version 22 (SPSS, Chicago, IL) was used. Statistical power calculations were performed using the R package genpwr on R version 4.3.2, thereby assessing statistical power across various genetic models including additive, dominant, recessive and 2df/unspecified model, comparing true and test genetic models [34]. The statistical power was evaluated in terms of odds ratio (2) and minor allele frequency (MAF).

Consistency of genotype frequencies with the Hardy–Weinberg equilibrium (HWE) was tested using a chi2 test on a contingency table of observed versus predicted genotype frequencies (*P* > 0.05).

## Results

### Study cohorts

Patients and clinical characteristics of both cohorts are presented in Table 1. No significant differences in patient characteristics between both cohorts were detected.

### Candidate SNP selection and allele frequency

Table S1 illustrates variants of the selected candidate genes previously reported to be associated with infectious events. All 11 candidate SNPs in 6 genes (*TLR2, TLR4, Dectin-1, DC-SIGN, PTX3, L-Ficolin*) have been described earlier to contribute to infectious events such as sepsis, pneumonia and IFD [5, 7–10, 12, 16, 25, 35–37]. Distribution of the allele frequencies is provided in Table S2. Notably, distribution of allele frequencies in AML patients were comparable to that in healthy individuals [38]. Selected patient samples were validated by Sanger sequencing and results of the TaqMan assay could be confirmed (Table S2). The statistical power for additive, dominant, recessive and unspecified genetic model was calculated for detecting an odds ratio of 2 and a MAF ranging from 0 to 40% (Fig. S1).

Importantly, complete linkage disequilibrium was found between *PTX3* SNPs rs2305619 and rs1840680 (r^2^ = 1).

### Infectious events

Table 2 summarizes the frequency of infectious events including sepsis, pneumonia and IFD.

**Table 2:** Frequency of infectious events.

	Stratification cohort (*n* = 186) *n* (%)	Validation cohort (*n* = 138) *n* (%)	*p* value
Sepsis	104 (55.9)	83 (60.1)	0.52
Pneumonia	69 (37.1)	52 (37.7)	1.0
IFD^a^	48 (25.8)	39 (28.3)	0.71
IFD (fluconazole)	20/95 (21.1)	0 (0)	n.a.
IFD (posaconazole)	26/91 (28.6)	39 (28.3)	1.0

^a^Patients of the stratification cohort received fluconazole or posaconazole as indicated, all patients of the validation cohort received posaconazole prophylaxis only.

*IFD*, invasive fungal disease.

As patients in the stratification cohort received either fluconazole or posaconazole antifungal prophylaxis, whereas in the validation cohort only posaconazole was administered, a comparison of rates of IFD for both treatments was conducted. No differences in the rate of IFD were detected when adjusted to the choice of antifungal prophylaxis (Table 2).

### SNPs associated with susceptibility to infectious events in AML patients following induction chemotherapy

Genetic association of SNP alleles with septic events and pneumonia including IFD are provided in Table 3. By multiple logistic regression analyses the following SNPs were detected to be significantly related to the occurrence of infectious events within the stratification cohort (Table 3A): *TLR2* Arg753Gln (rs5743708), *Dectin-1* rs7309123, *DC-SIGN* (CD209) rs4804800, *DC-Sign* (CD209) rs7248637, *Ficolin 2* rs17514136, *Ficolin 2* rs17549193. For the validation cohort (Table 3B) *Dectin-1* Y238X rs16910526, *DC-SIGN* (CD209) rs4804800, *PTX3* rs3816527*, PTX3* rs2305619 and *Ficolin 2* rs17514136 have found to be associated with infectious events. One SNP was validated to be related to the occurrence of sepsis in both cohorts: *DC-SIGN* (CD209) rs4804800.

**Table 3:** Multivariate analysis of SNPs associated with the development of infectious events following induction chemotherapy.

A. Stratification cohort (*n* = 186)			
Genetic variable	Sepsis; OR (95% CI), *p* value	Pneumonia; OR (95% CI), *p* value	IFD ; OR (95% CI), *p* value
TLR4 399 rs4986791	0.70 (0.28–1.75) 0.439	1.17 (0.45–3.04) 0.754	1.38 (0.47–4.11) 0.562
TLR2 Arg753Gln rs5743708	4.26 (0.91–20.0) 0.067	**9.52 (1.96**–**46.23),** **0.005**	**4.27 (1.23**–**14.81),** **0.02**
Dectin-1 Y238X rs 16910526	1.81 (0.66–4.99) 0.252	0.79 (0.28–2-27) 0.673	0.79 (0.24–2.57) 0.688
Dectin-1 rs7309123 G/G genotype	1.55 (0.78–3.07) 0.208	**2.33 (1.14**–**4.76),** **0.020**	**2.33 (1.12**–**4.86),** **0.024**
Dectin-1 rs7309123 G/G + C/G genotype	**2.84 (1.42–5.67), 0.003**	**3.17 (1.43–7.04),** **0.004**	**2.81 (1.15–6.87), 0.024**
DC-SIGN rs4804800 G/A + G/G genotype	**2.37 (1.19–4.72), 0.015**	**2.26 (1.15–4.46),** **0.019**	1.93 (0.94–3.98) 0.075
DC-SIGN rs7248637 A/G + A/A genotype	1.94 (0.91–4.14) 0.852	1.91 (0.90–4.04) 0.913	1.91 (0.87–4.19) 0.114
PTX3 rs3816527 A/A + A/C genotype	0.89 (0.49–1.61) 0.693	0.98 (0.52–1.86) 0.961	0.98 (0.49–1.98) 0.970
PTX3 rs2305619 A/A + G/A genotype	1.29 (0.66–2.53) 0.457	0.91 (0.45–1.87) 0.806	1.05 (0.47–2.34) 0.910
PTX3 rs1840680 A/A + G/A genotype	1.36 (0.63–2.94) 0.433	0.93 (0.41–3.0) 0.855	1.04 (0.42–2.58) 0.941
Ficolin 2 rs17514136 G/G genotype	1.44 (0.57–3.62) 0.439	**2.67 (1.04–6.82),** **0.040**	0.97 (0.35–2.73) 0.955
Ficolin 2 rs17514136 G/G + A/G genotype	**1.93 (1.06–3.51), 0.032**	1.26 (0.68–2.33) 0.471	0.95 (0.48–1.86) 0.872
Ficolin 2 rs17514136 A/A + G/A genotype	0.71 (0.28–1.82) 0.472	0.84 (0.56–3.64) 0.365	1.03 (0.37–2.90) 0.955
Ficolin 2 rs17549193 T/T genotype	1.44 (0.57–3.62) 0.439	**2.83 (1.10–7.23),** **0.030**	1.03 (0.37–2.90) 0.952
Ficolin 2 rs17549193 T/T + C/T genotype	**1.92 (1.1–3.45),** **0.031**	1.27 (0.68–2.37) 0.449	1.03 (0.52–2.03) 0.943

B. Validation cohort *(n* = 138)			
TLR4 399 rs4986791	0.43 (0.16–1.14) 0.09	0.55 (0.19–1.62) 0.276	0.26 (0.06–1.19) 0.082
TLR2 Arg753Gln rs5743708	1.17 (0.33–4.22) 0.805	0.94 (0.26–3.38) 0.925	1.50 (0.41–5.45) 0.54
Dectin-1 Y238X rs 16910526	1.94 (0.75–5.03) 0.173	2.05 (0.85–4.94) 0.110	**2.88 (1.17–7.08), 0.021**
Dectin-1 rs7309123 G/G genotype	1.03 (0.44–2.41) 0.945	1.09 (0.47–2.55) 0.844	1.26 (0.51–3.10) 0.610
Dectin-1 rs7309123 G/G + C/G genotype	1.46 (0.72–2.98) 0.296	1.16 (0.56–2.40) 0.689	1.52 (0.67–2.41) 0.310
DC-SIGN rs4804800 G/A	**3.49 (1.40–8.71), 0.07**	1.57 (0.72–3.41) 0.258	1.02 (0.44–2.39) 0.962
DC-SIGN rs7248637 A/G genotype	1.73 (0.75–3.98) 0.202	1.53 (0.69–3.39) 0.292	0.76 (0.31–1.88) 0.557
PTX3 rs3816527 C/C + A/C genotype	**3.37 (1.61–7.06), 0.001**	**4.54 (1.09–10.86),** **0.001**	**4.89 (1.76–13.58), 0.002**
PTX3 rs2305619 A/A + A/C genotype	**2.88 (1.25–6.61), 0.013**	**7.48 (2.14–26.13),** **0.002**	**7.30 (1.65–32.33), 0.009**
PTX3 rs1840680 A/A + G/A genotype	**2.88 (1.25–6.61), 0.013**	**7.48 (2.14–26.13),** **0.002**	**7.30 (1.65–32.33), 0.009**
Ficolin 2 rs17514136 G/G genotype	0.56 (0.73–1.59) 0.295	0.53 (0.10–2.75) 0.452	0.84 (0.16–4.34) 0.833
Ficolin 2 rs17514136 G/G + A/G genotype	0.99 (0.49–1.99) 0.977	1.73 (0.86–3.49) 0.126	1.47 (0.68–3.04) 0.344
Ficolin 2 rs17514136 A/A + G/A genotype	**11.96 (1.43–100.16),** **0.022**	1.86 (0.36–9.66) 0.452	1.19 (0.23–6.18) 0.833
Ficolin 2 rs17549193 T/T genotype	0.31 (0.73–1.28) 0.115	1.35 (0.35–5.27) 0.666	1.29 (0.31–5.44) 0.727
Ficolin 2 rs17549193 T/T + C/T genotype	0.99 (0.49–1.98) 0.977	1.73 (0.86–3.49) 0.126	1.44 (0.68–3.04) 0.344

Bold values identify statistical significance.

### SNPs associated with IFD development

Due to the importance of IFD as a significant cause of infectious morbidity and mortality in AML patients receiving induction chemotherapy, the current study focused on previously reported SNPs contributing to fungal pneumonia. Considering both cohorts separately, the following genetic associations were observed to be significantly associated by multivariate analysis in the stratification cohort: *TLR2* Arg753Gln (rs5743708), *Dectin-1* rs73909123 and in the validation cohort: *Dectin-1* Y238X rs16910526, *PTX3* rs3816527, *PTX3* rs2305619, *PTX* rs1840680 (Table 3).

## Discussion

Multiple SNPs of the innate immune system have been suspected to alter the susceptibility to infectious events in patients with AML. Most of the studies were performed within the setting of alloHSCT which might not represent an ideal model of patients´ susceptibility due to varying involvement of donor-recipient immune system interactions. This study aimed to investigate infectious events in AML patients undergoing intensive induction chemotherapy, where the innate immune system´s capacity to combat infections is crucial due to prolonged neutropenia. Certain genetic risk factors, particularly SNPs, within the host´s genetic code associated with the innate immune response have been linked to increased risk of developing IFD and other types of infection [7, 8, 39, 40]. As consistency across studies regarding the identified SNPs associated with infectious events remains critical, a retrospective analysis of two distinct cohorts of AML patients was conducted to provide validation. Hence, this study serves as a validation for previously published findings while also broadening the scope through the inclusion of additional SNPs and investigating their association with infectious complications following induction chemotherapy. Both in the stratification cohort and the validation cohort only patients with initial diagnosis of AML and those who received intensive induction chemotherapy were eligible for analysis. Patients’ characteristics were equally distributed and frequencies of infectious events like sepsis and IFD did not differ statistically. Due to the more recent approval of posaconazole, half of the patients in the stratification cohort received fluconazole. Although not statistically significant a slight increase in IFD was diagnosed within the posaconazole group of the stratification cohort which might be explained by more sensitive radiographic methods during recent years. Consistent with the finding of the overall stratification cohort, analyzing only the posaconazole patients of that cohort revealed no significant association between the development of IFD and the presence of PTX3 or Dectin Y238X. This contrasts the validation cohort where SNPs of both genes have been found to be associated with the occurrence of IFD. The *PTX3* gene SNPs rs2305619/rs1840680 and rs3816527 were linked to the development of IFD within the alloHSCT setting [9, 10]. Of note, those studies showed the GG genotype of the *PTX3* rs2305619 associated with a higher risk of IFD. For the rs3816527 of the *PTX3* gene Cunha et al. found the AA donor genotype more frequently in patients with IFD following alloHSCT [10]. In our study, the AA + AG genotype for *PTX3* rs2305619/rs1840680 and CC + AC genotype for *PTX3* rs3816527 were associated with the development of IFD. Due to these contradictory results, Sanger sequencing was performed and confirmed the TaqMan genotyping assay results. Another study reports on the significant interaction of the *PTX3* rs2305619 and rs3816527 SNPs in AML patients and pre-existing neutropenia when developing IFD [41]. By stratifying to absolute neutrophil count it was shown that homozygosity for the minor allele of both *PTX3* SNPs was an independent predictor of invasive mold infections. In accordance to these data, another report of non-AML patients was able to demonstrate that patients with chronic obstructive pulmonary disease (COPD) homozygote for the minor allele (A) of SNP rs1840690 harbored a higher risk for IFD [16]. The authors did also reveal significantly higher plasma *PTX3* levels in COPD patients with IFD compared to controls. In addition, patients with the AA genotype had lower plasma *PTX3* levels compared to those with the AG and GG genotype hypothesizing exhausted *PTX3* storage leading to an increased susceptibility for IFD. A more recent study involving non-hematological patients assessed the relationship between the *PTX3* rs1840680 polymorphism and its impact on the clinical course of coronavirus disease 2019 (COVID-19) [42]. Macrophage activation syndrome (MAS) as the main cause of morbidity and mortality in COVID-19 patients was significantly more frequent in patients with the AA genotype of the *PTX3* rs1840680 SNP. In contrast, the AG genotype might play a protective role in developing MAS.

A SNP of the *DC-SIGN* gene, rs4804800, was identified to be associated with the occurrence of sepsis in both cohorts. While this genetic variant has been described earlier to be associated with the development of IFD in hematological patients, here, an association has been demonstrated within two homogenous AML patient cohorts. Patients carrying the G (AG and GG) allele showed a 2.37 (1.19–4.72) and a 3.49 (1.40–8.71) risk of developing sepsis within the stratification cohort and the validation cohort, respectively.

In summary, our study shows a statistically significant association between SNPs of the *PTX3* (rs2305619/rs1840680, rs3816527) and *Dectin-1* (Y238X, rs16910526) genes and the development of IFD intensive induction chemotherapy induced neutropenia in AML patients. Furthermore, SNPs of *Ficolin 2* gene could have been identified to contribute to septic events.

A single SNP, rs4804800, of the *DC-SIGN* gene could be detected and validated in a multivariate analysis of having a statistically significant impact on the occurrence of sepsis in both cohorts.

The key strength of the study is the presence of a second independent cohort in which the selected SNPs were also investigated. Additionally, in contrast to other studies, a homogenous non-alloHSCT setting of AML patients receiving cytarabine and anthracycline based induction chemotherapy was analyzed. Although major limitations of earlier studies have been addressed by performing an analysis of the identified SNPs in a second cohort, there are certainly some limitations. Most of the SNPs identified as being associated with infectious events in AML patients were found in only one cohort, not both. These conflicting results regarding the differences between both cohorts in association with specific SNPs and infectious events might be at least partially explained by the small sample sizes. Those inconsistencies in the association of specific SNPs with the risk of infectious events, such as IFD, need to be resolved through large scale, multi-center trials of promising candidate genes. Another explanation might be the different administration of induction chemotherapy. In the stratification cohort, induction therapy was typically administered as a single induction according to the OSHO protocol, whereas in the validation cohort, a double induction therapy according to the 7 + 3 regimen was applied. Due to the longer duration of neutropenia after double induction, PTX3 might play a more important role in the occurrence of IFD in the validation cohort compared to the stratification cohort. Specifically, for PTX3, it has been shown that the frequency of IFD increases over time.

Taken together, infectious diseases such as IFD remain a major cause of morbidity and mortality in AML patients during neutropenia following induction chemotherapy. Patients at high risk can be identified by clinical risk assessment and by combined biomarker screening. Through the combination of clinical and genetic risk evaluation physicians might predict the risk of developing IFD more accurately in the future. Screening for individual host specific SNPs as part of the clinical workup on admission might direct decisions regarding antifungal prophylaxis and treatment.

### Supplementary information

Supplemental Material of Validating genetic variants in innate immunity linked to infectious events in acute myeloid leukemia post-induction chemotherapy

## Data Availability

The data supporting the findings of this study are available within the article and its supplementary materials. Additional data that support the findings of this study are freely available to any researcher wishing to use them for non-commercial purposes from the corresponding author, Ulf Schnetzke, upon reasonable request. Requests for data should include a detailed proposal, and data access will be granted in accordance with institutional guidelines and data sharing policies.
