# Supplementary material for: Validating genetic variants in innate immunity linked to infectious events in acute myeloid leukemia post-induction chemotherapy
Source: Genes Immun. 2024 Jul 9;25(4):317–23. doi: 10.1038/s41435-024-00285-4 (PMC11327101; doi:10.1038/s41435-024-00285-4)
Supplement: Supplementary file 1 — Supplemental Material of Validating genetic variants in innate immunity linked to infectious events in acute myeloid leukemia post-induction chemotherapy [file 41435_2024_285_MOESM1_ESM.docx]

**Table S1: Genetic variants of candidate genes previously associated with infectious events**

| **Gene** | **SNP** | **infectious event** | **reference** | **group** | **N** | **alleles § /**  **risk allele/gentotype** | **MAF** |
| --- | --- | --- | --- | --- | --- | --- | --- |
| TLR2 | rs5743708 | Pneumonia, IFD | ^8^ | AML, induction therapy | 186 | G/A  A | 0.01 |
| TLR4 | rs4986791 | IFD | ^39^ | HCT | 366 | C/T  T | 0.04 |
| Dectin-1 | rs16910526  rs 7309123 | IFD  IFD  IFD | ^14^  ^15^  ^25^ | HCT  non-HCT  HCT and non-HCT | 205  21  182 | T/G  G  C/G  G | 0.04  0.28 |
| DC-SIGN | rs4804800  rs7248637 | IFD  IFD | ^25^  ^25^ | HCT and non-HCT  HCT and non-HCT | 182  182 | A/G  G  G/A  A | 0.22  0.23 |
| PTX3 | rs3816527  rs2305619† | IFD  IFD | ^10^  ^40^  ^10^  ^40^  ^37^ | HCT  non-HCT  HCT  non-HCT  HCT | 330  185  330  185  251 | A/C  A  A/C  CC  G/A  G  G/A  AA  G/A  GG and AA | 0.29  0.44 |
| Ficolin 2 | rs17514136  rs17549193 | Streptococcal infection  Schistosomoasis  Blood stream infection | ^42^  ^43^  ^44^ | non-hematological  non-hematological  non-hematological | 122  168  81 | A/G  A  A/G  A  C/T  T | 0.19  0.25 |

**Abbreviations:** IFD, invasive fungal disease; AML, acute myeloid leukemia; MAF, minor allele frequency; HCT, hematopoietic stem cell transplantation

§ major/minor allele

† complete linkage disequilibrium to rs1840680

**Table S2: Distribution of allele frequencies**

| Gene  SNP  alleles* | Wild-type  stratification cohort n (%)  validation cohort n (%) | Heterozygous  stratification cohort n (%)  validation cohort n (%) | Homozygous  stratification cohort n (%)  validation cohort n (%) |
| --- | --- | --- | --- |
| TLR4 Thr399Ile  rs4986791  C/T | CC  164 (88.2)  120 (87) | CT  22 (11.8)  18 (13) | TT  0 (0)  0 (0) |
| TLR2 Arg753Gln  rs5743708  G/A | GG  174 (93.5)  127 (92) | GA  12 (6.5)  11 (8) | AA  0 (0)  0 (0) |
| Dectin-1 Y238X  rs16910526  T/G | TT  167 (89.9)  107 (77.5) | TG  19 (10.1)  25 (18.1); 6 (4.3) n.d. | GG  0 (0)  0 (0) |
| Dectin-1  rs72309123  C/G | CC  53 (28.5)  47 (34.1) | CG  87 (46.8)  63 (45.7) | GG  46 (24.7)  28 (20.2) |
| DC-SIGN  rs4804800  A/G | AA  135 (72.6)  103 (74.6) | AG  45 (24.2)  35 (25.4) | GG  6 (3.2)  0 (0) |
| DC-SIGN  rs7248637  G/A | GG  148 (79.6)  105 (76.1) | AG  32 (17.2)  33 (23.9) | AA  6 (3.2)  0 (0) |
| PTX3  rs3816527  A/C | AA  71 (38.2)  46 (33.3) | AC  76 (40.9)  73 (52.9) | CC  39 (21.0)  18 (13), 1 (0.7) n.d. |
| PTX3  rs2305619 ^§^  G/A | GG  63 (33.9)  30 (21.7) | GA  78 (41.9)  83 (60.1) | AA  45 (24.2)  25 (18.1) |
| Ficolin 2  rs17514136  A/G | AA  86 (46.2)  83 (60.1) | AG  78 (41.9)  47 (34.1) | GG  22 (11.8)  8 (5.8) |
| Ficolin 2  rs17549193  C/T | CC  81 (43.5)  82 (59.4) | CT  83 (44.6)  47 (34.1) | TT  22 (11.9)  9 (6.5) |

**Abbreviations:** n.d, no data

*major/minor

§ complete linkage disequilibrium to rs1840680

**Table S3: Selective validation of TaqMan genotyping by Sanger sequencing**

| SNP Assay | Confidence | Allele 1 | Allele 2 | Call | Genotype |
| --- | --- | --- | --- | --- | --- |
| rs3816527 PTX-3 | 0,98866077 | 3,29476818 | 3,70203149 | VIC_A/VIC_A | A/A |
| rs2305619 PTX-3 | 0,98866077 | 0,59093481 | 6,21192479 | FAM_G/FAM_G | G/G |
| rs3816527 PTX-3 | 0,98866077 | 1,66167593 | 7,51065427 | FAM_C/FAM_C | C/C |
| rs2305619 PTX-3 | 0,98866077 | 3,77934033 | 0,92117071 | VIC_A/VIC_A | A/A |
| rs3816527 PTX-3 | 0,98866077 | 1,63272586 | 7,32105958 | FAM_C/FAM_C | C/C |
| rs2305619 PTX-3 | 0,98866077 | 3,73166347 | 0,90685213 | VIC_A/VIC_A | A/A |
| rs3816527 PTX-3 | 0,98866077 | 3,09428853 | 3,49724379 | VIC_A/VIC_A | A/A |
| rs2305619 PTX-3 | 0,98866077 | 0,58725929 | 6,00434017 | FAM_G/FAM_G | G/G |

**Figure S1:**

**A)**


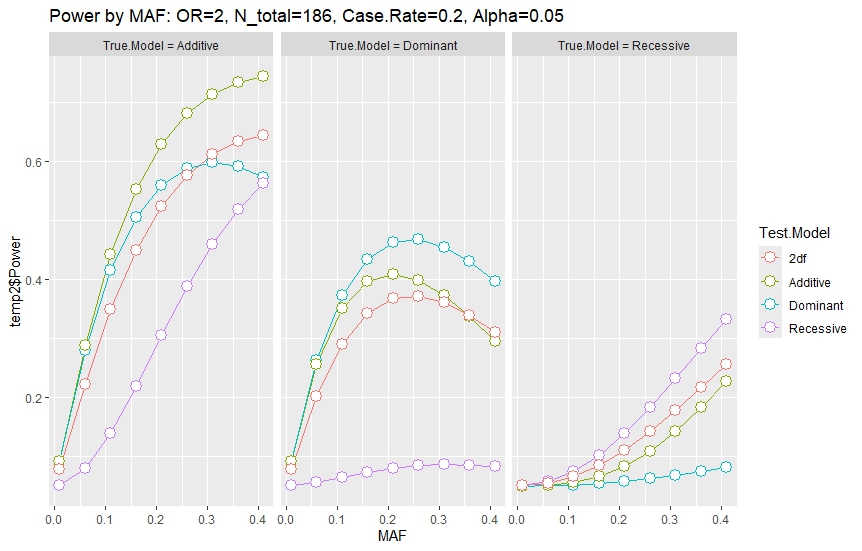


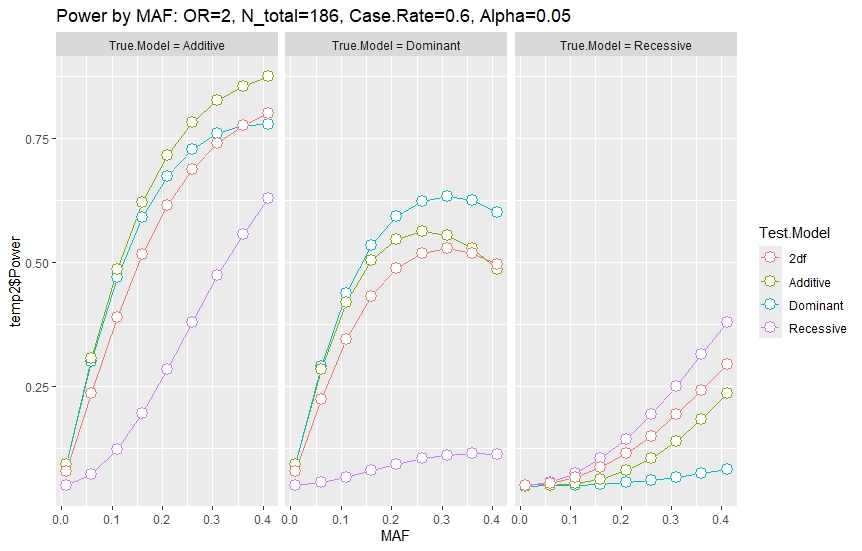


Suppl. Figure 1A (stratification cohort, n = 186): Comparison-wise power (y-axis) for varying minor allele frequencies (MAF; x-axis) and different models testable for genetic associations (colors) for a fixed sample size of n=186, a case proportion of 20% (upper panel) or 60% (lower panel) at a significance level α=.05 to detect a genetic effect (odds ratio (OR)) of 2. Plots were generated using the R package genpwr in R version 4.3.2.

**B)**


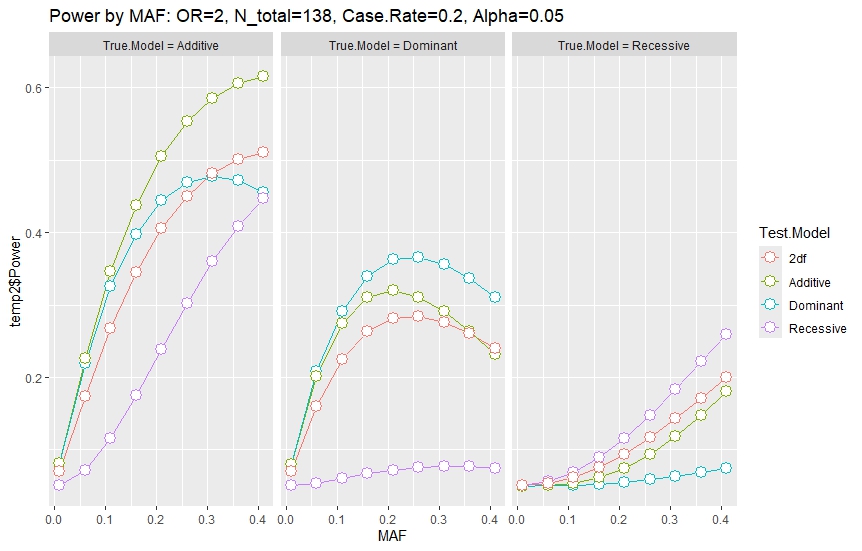


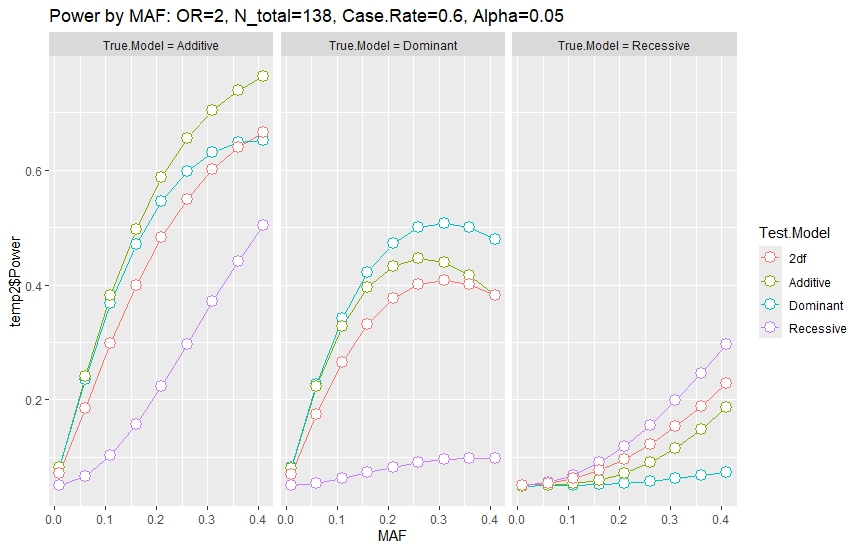


Suppl. Figure 1B (validation cohort, n = 138): Comparison-wise power (y-axis) for varying minor allele frequencies (MAF; x-axis) and different models testable for genetic associations (colors) for a fixed sample size of n=138, a case proportion of 20% (upper panel) or 60% (lower panel) at a significance level α=.05 to detect a genetic effect (odds ratio (OR)) of 2. Plots were generated using the R package genpwr in R version 4.3.2.
